# Reduced Culture Temperature Differentially Affects Expression and Biophysical Properties of Monoclonal Antibody Variants

**DOI:** 10.3390/antib3030253

**Published:** 2014-08-29

**Authors:** Megan Mason, Bernadette Sweeney, Katharine Cain, Paul Stephens, Susan T. Sharfstein

**Affiliations:** 1 Department of Chemical Engineering and Center for Interdisciplinary Studies, Rensselaer Polytechnic Institute, Troy, NY 12180, USA;; 2 Protein Expression and Purification Group, UCB Celltech, Slough, SL1 4EN, Berkshire, UK;; 3 SUNY College of Nanoscale Science and Engineering, Albany, NY 12203, USA

**Keywords:** Chinese hamster ovary cells, monoclonal antibodies, protein stability, temperature shift, antibody engineering

## Abstract

Reduced culture temperature is an increasingly popular practice to improve recombinant protein yields in CHO cells. Recent studies have attributed the enhancement of protein titers at sub-physiological temperatures to increased mRNA levels as well as extended stationary phase. We observed that reducing the culture temperature arrested cell growth, prolonged viability, and increased cell size. However, the reduced culture temperature had a differential effect on protein and mRNA expression of closely related antibody mutants from stable cell lines. The highly expressing mutant (Ala) exhibited similar or decreased specific productivity and decreased volumetric productivity over the culture lifetime at 32 °C compared to 37 °C. In contrast, the specific and volumetric productivity of the poorly expressing mutant (Gly) was enhanced at the lower culture temperature. The difference in specific productivity was reflected in the amounts of heavy- and light-chain mRNA. Analysis of the secondary and tertiary configurations of the purified antibodies by circular dichroism revealed fundamental structural differences imposed by the Ala to Gly mutation as well as reduced culture temperature. We propose that the effect of reduced culture temperature on expression is protein-dependent; protein folding fidelity and assembly is improved at lower temperatures, enhancing the expression of proteins that have a propensity to misfold.

## 1. Introduction

Mammalian cell lines, such as Chinese hamster ovary (CHO) cells, are the industry standard for production of recombinant therapeutic proteins, including monoclonal antibodies, hormones, blood factors, and enzymes [1]. The requirement for high-producing cell lines to meet the increasing demand for biopharmaceuticals has driven the evolution of mammalian expression systems. Development of novel expression vectors, robust cell-culture media, stringent cell-line selection strategies, and efficient production processes have pushed maximum achievable titers well into the g/L range [2,3]. Reduction of cultivation temperature is becoming a commonly used method to enhance recombinant protein expression in mammalian cells [4,5]; it avoids the use of potentially toxic additives, expensive equipment, and can be applied in parallel with other techniques.

The general response of mammalian cells to cold is thought to involve a combination of responses regulated by cold-shock proteins (CSPs) including reduced metabolism, cell-cycle arrest, predominately in the G_1_ phase, increased viability for prolonged periods of time, and transcriptional/translational attenuation [4,6]. Intuitively, the repression of the gene expression machinery should decrease exogenous protein production. To explain why the opposite effect is often observed, reports suggest increased RNA half-life, increased transcription of certain target genes through specific binding sites within their promoter regions, alternative mRNA splicing, and CSP-mediated Internal Ribosome Entry Segments (IRESs) in certain mRNAs may be responsible for increased gene expression at sub-physiological temperatures [6]. Recently the Smales’ group showed that cap-dependent mRNA translation is not severely attenuated in CHOK1 cells when the culture temperature is shifted from 37 °C to 32 °C [7]. They suggested the increase in protein expression was due to a combination of increased mRNA half-life and improved fidelity of protein folding and post-translational events at 32 °C.

Although the majority of studies indicate mild hypothermia improves gene expression, there are also reports where decreasing culture temperature had little or no effect on expression [8–11]. The cold-shock response in mammalian cells is poorly understood, and further study using a wide range of proteins and cell lines is critical to gaining insight to the biological mechanisms involved in reduced temperature cell culture. In this study, we examined the effect of culture temperature on expression of a pair of antibodies that differ by a single amino acid in the heavy chain variable-region framework. We also determined the effect of culture temperature on the biophysical properties of the secreted antibody products in attempt to elucidate how reduced culture temperature can improve recombinant protein expression and identify the cellular mechanisms involved in this enhancement.

## 2. Results and Discussion

### 2.1. Transient Expression

**Figure 1.**
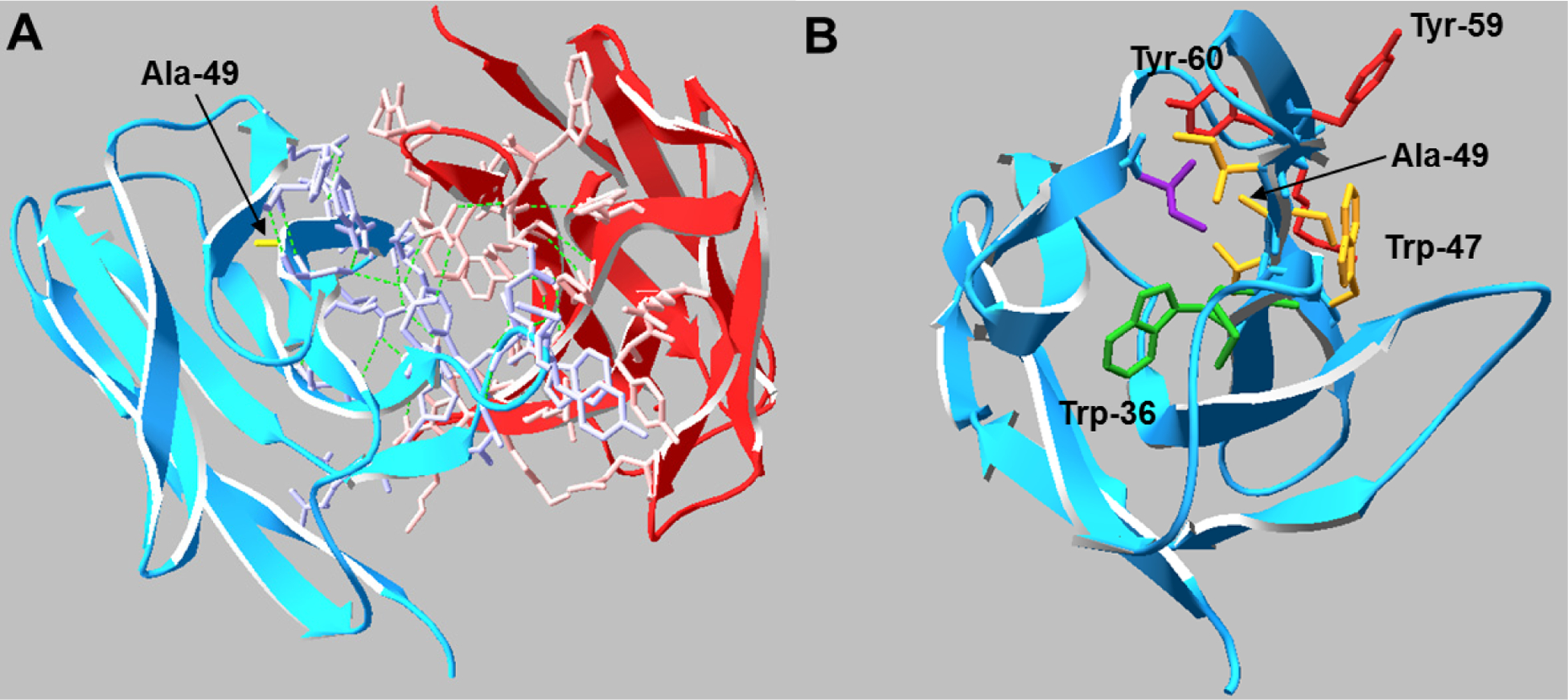
(**A**) Crystal structure of the V_H_/V_L_ interactions in the Ala Fab domain. Residues that participate in the HC (blue) /LC (red) interface are shown in wire representation and the Ala-49 side chain is labeled (yellow). Hydrogen bonds are shown as green dashed lines. (**B**) Top view of the V_H_ domain. Residues that may be conformationally influenced by Ala-49 (<5 Å distance) are shown in wire representation. Side chains sharing the same color reside on the same β-strand.

**Figure 2.**
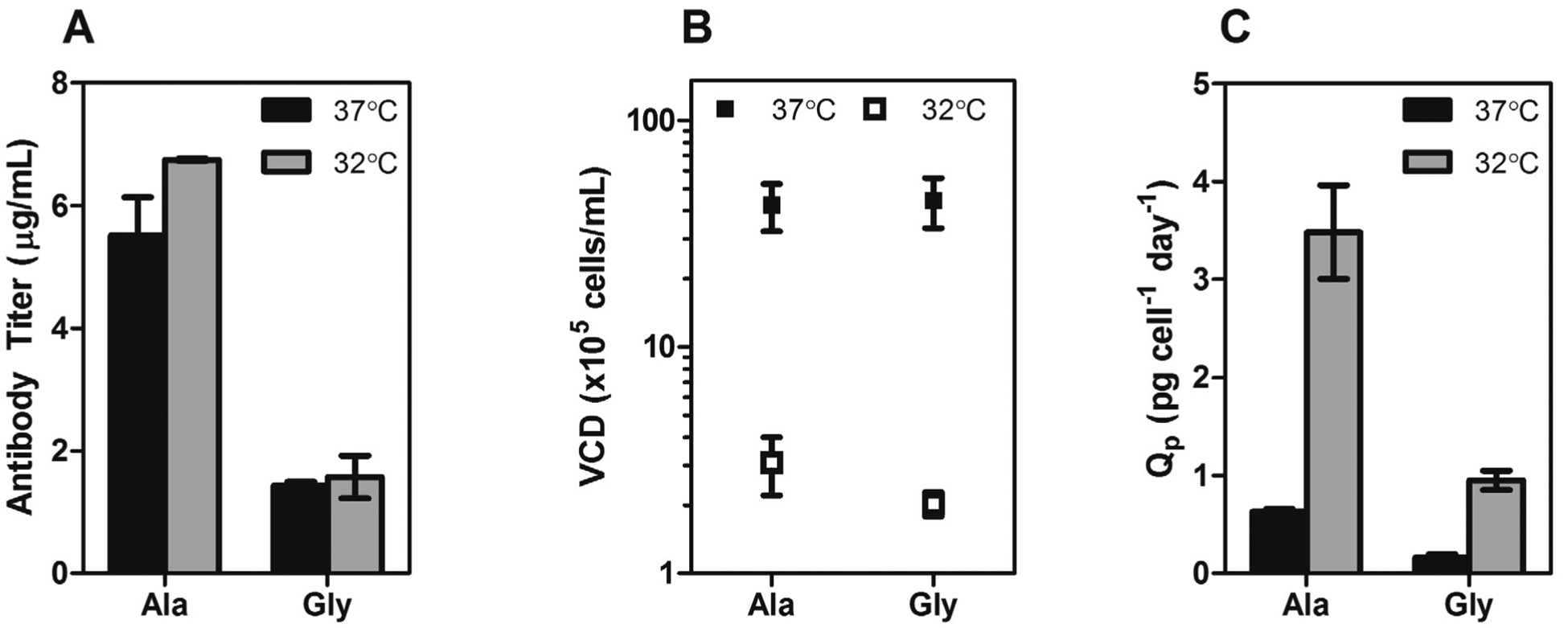
(**A**) Transient expression titers of Ala and Gly antibodies after a 5-day incubation at 37 °C (black bars) or 32 °C (gray bars). (**B**) The viable cell density (VCD) of the Ala and Gly cultures at 37 °C (■) and 32 °C (□). (**C**) Specific productivities of Ala and Gly antibodies at 37 °C (black bars) or 32 °C (gray bars). The error bars represent ± 1 standard deviation from the mean of experimental replicates (n = 2).

A pair of IgG_4_ antibodies was previously identified [12] whereby an Ala to Gly mutation at Kabat position 49 resulted in a four-fold decrease in transient CHO expression. The Gly is the “naturally preferred” amino acid at this position and is present in ~50% of human antibody sequences with Ala and Ser equally contributing to the other 50% [13]. The mutation lies within the heavy chain variable framework, two residues before the CDR2 loop. Analysis of the Fab crystal structure suggests the side chain of any residue in Kabat position 49 may play a critical role in stabilizing the hydrophobic core of the V_H_ domain as well as maintaining the conformation of adjacent residues which comprise part of the V_H_/V_L_ interface (Figure 1). To determine if mild hypothermia alters the innate differential expression of this antibody pair, CHO-S cells were transiently transfected with Ala or Gly double gene vectors (DGV, described in Section 3.2) and immediately incubated at 37 °C or 32 °C for five days. The Ala variant exhibited four-fold higher monoclonal antibody (mAb) expression than the Gly variant at 37 °C and 32 °C (Figure 2a). Culturing the cells at 32 °C gave a slight improvement in mAb titer for both constructs (~1.2-fold), however the total viable cell densities for the cultures at 37 °C were at least 10-fold higher than the cultures at 32 °C (Figure 2b), indicating that the average specific productivity was higher for the low temperature cultures (Figure 2c).

Exposing CHO cultures to sub-physiological temperatures is known to arrest cells primarily in the G0/G1 phase and can correlate with high levels of biosynthetic activity [5,10,11,14]. The growth arrest of the 32 °C cultures over the entirety of the five day incubation is in agreement with this observation. A study by Wurm and coworkers found that performing a temperature shift from 37 °C to 31 °C at 0–4 h post-transfection resulted in the highest transient expression levels of an IgG in CHO cells [14]. They could not attribute the increase in expression to a specific mechanism but suggested DNA uptake into the nucleus, DNA/mRNA stability, transcription, translation, and/or protein modification and transport may be responsible. Here, we observed that an immediate shift to 32 °C resulted in high apparent specific productivity, but only a marginal increase in volumetric productivity. Because the Amaxa Nucelofector method allows the transgenic DNA to directly enter the nucleus, the cells are capable of expression immediately and cell division is not required. This could suggest that the inhibition of cell division at 32 °C prevents dilution of intracellular recombinant DNA; in the 37 °C cultures the same amount of DNA is spread throughout a larger number of cells resulting in constant volumetric productivity, but decreased apparent specific productivity.

### 2.2. Stable Expression

**Figure 3.**
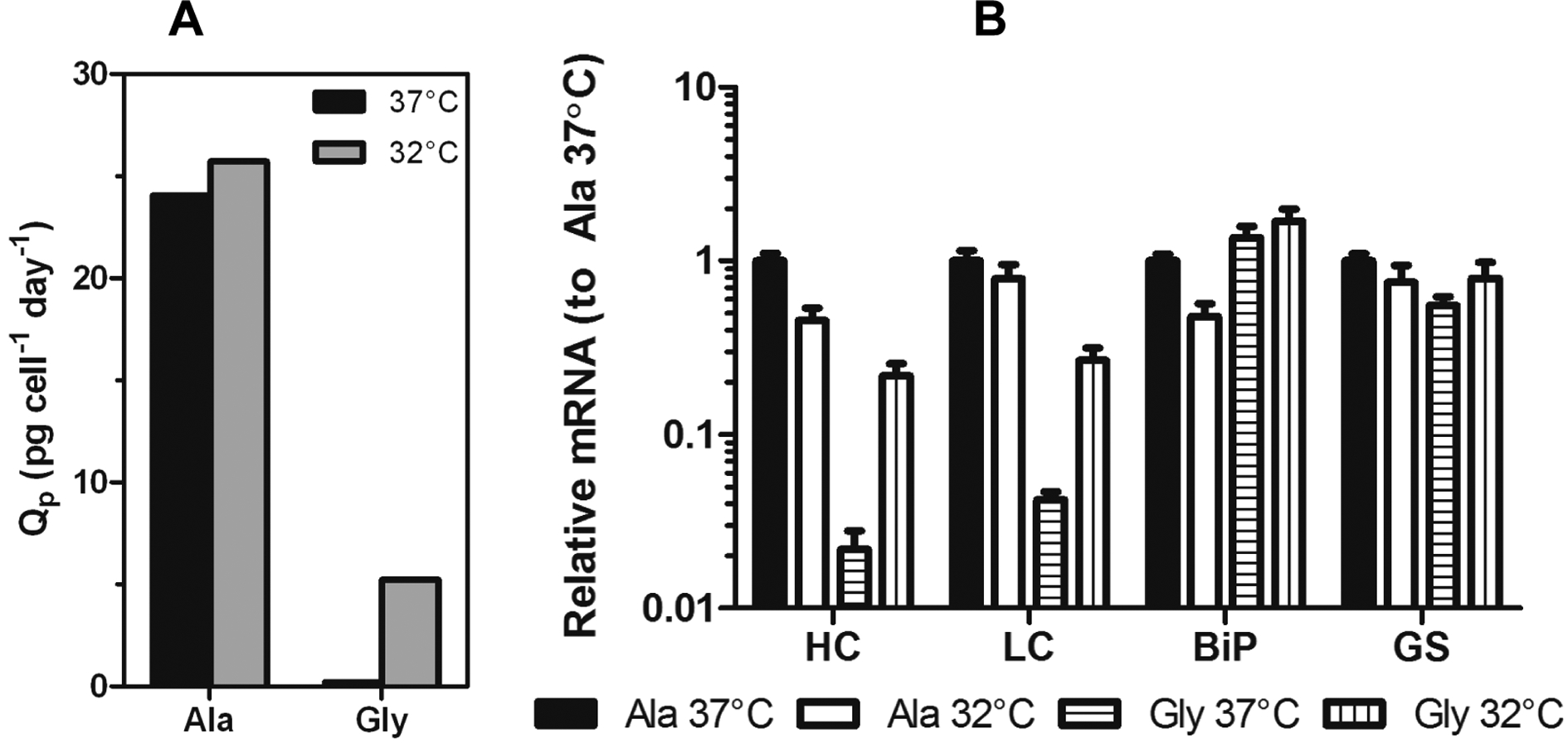
(**A**) Specific productivity (Q_p_) of Ala-138 and Gly-26 mAb-producing clones in the mid-exponential growth phase at 37 °C, days 3–6 (black bars) and 32 °C, days 6–9 (gray bars). (**B**) Expression of HC, LC, BiP, and GS mRNA in Ala-138 and Gly-26 clones on day 4 at 37 °C or 32 °C incubation temperature. The mRNA levels for each gene were calibrated to Ala-138 at 37 °C. The error bars represent ±1 standard deviation of technical replicates (n = 3). 37 °C cultures were compared with 32 °C using a paired t-test (GraphPad Prism 5.03) and found to be statistically different (*p* = 0.0229 for Ala cultures and *p* = 0.0038 for Gly cultures).

To assess if specific productivity of stable systems is improved under mild hypothermia, multiple clonal cell lines stably expressing identical constructs to those used in the transient study were established as previously described [12]. Cell lines were subcultured in the presence of 50 μM MSX and inoculated into shake flasks at 3 × 10^5^ cells/mL followed by immediate incubation at either 37 °C or 32 °C. Samples were removed periodically to measure mAb titer and cell number. The initial experiments were performed on high expressing Ala (Ala-138, high expressing variant) and Gly (Gly-26, low expressing variant) clones. These cell lines exhibited a 100-fold difference in expression when cultured at 37 °C. Culturing the clones at 32 °C reduced the difference in expression between the Ala and Gly cell lines to 20-fold due to a 2-fold decrease in Ala-138 titer and a 10-fold increase in Gly-26 titer. When the decrease in growth rate was taken into account, the Ala specific productivity remained largely unchanged, whereas the Gly specific productivity increased 25-fold (Figure 3a).

Samples from mid-exponential phase Ala-138 and Gly-26 clones cultured at 37 °C and 32 °C were analyzed using qRT-PCR to determine if decreased culture incubation temperature correlated with an increase in transgene mRNA. Decreasing culture temperature reduced the Ala HC and LC mRNA levels, whereas it resulted in a 10-fold increase in Gly HC and LC mRNA (Figure 3b). GAPDH was used as an endogenous control. Similar, though less significant, changes were seen in immunoglobulin binding protein (BiP) and glutamine synthetase (GS). The decrease in Ala mRNA and increase in Gly mRNA contradicts previous studies attributing increased mRNA half-life and transcription as the primary mechanisms responsible for improvements in recombinant gene expression at reduced temperature. If mRNA-related mechanisms were the sole contributors to increased expression at reduced temperatures, then a universal increase in message for both constructs should be observed.

**Figure 4.**
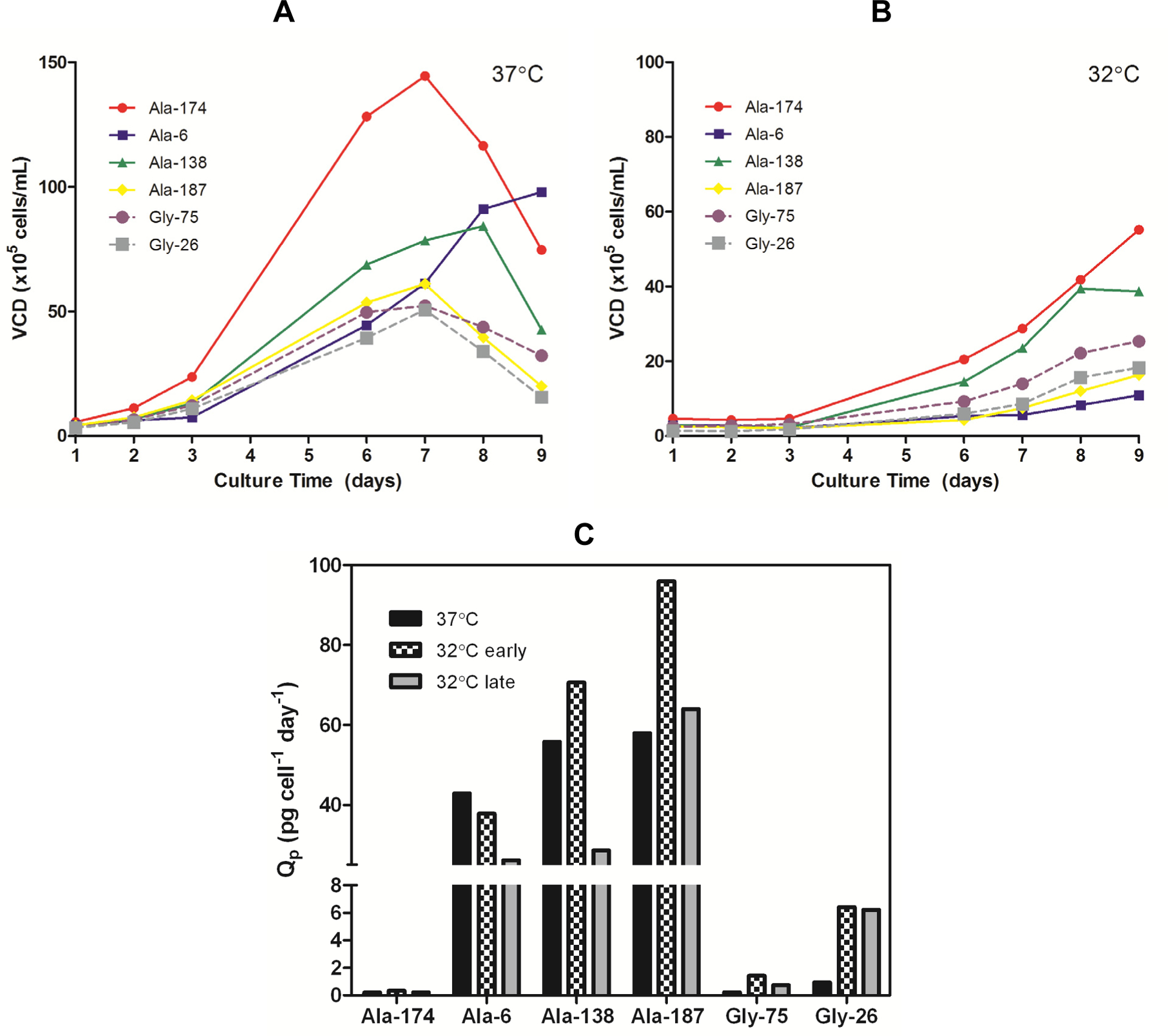
(**A**) Viable cell density (VCD) profiles for Ala and Gly clones grown at 37 °C in serum-free batch culture. (**B**) VCD profiles for Ala and Gly clones grown at 32 °C in serum-free batch culture. (**C**) Specific productivity (Q_p_) of Ala and Gly clones in mid-exponential growth phase at 37 °C (days 3–6) and 32 °C (early phase: days 3–6; late phase: days 7–8).

Clonal variation in recombinant protein expression is typically attributed to integration events that affect the ability of the DNA to be readily transcribed and therefore, change the level of transgene mRNA available for translation. A study by Yoon *et al.* [15] showed that the degree of Q_p_ enhancement under reduced temperature conditions varied between clones and that the enhancement decreased with increasing gene amplification. To ensure the differential effect of temperature on the expression of the mAb variants was not due to clonal variation, several clones (previously described by Mason *et al.* [12]) exhibiting different growth and expression profiles were analyzed in parallel. All clones exhibited the same differential effect of temperature on expression that was previously observed; all Ala clones exhibited compromised mAb expression when cultured at lower temperatures, whereas the Gly clones benefited (Table 1). Comparison of the growth curves for all clones at 37 °C *versus* 32 °C (Figure 4a,b) showed the cells cultured at the lower temperature had an extended lag phase up to three days, followed by a dynamic exponential growth phase as the cells adapted to the lower temperature, and finally a plateau in the stationary phase around day nine. The change in growth rate during the exponential phase, presumably due to the cells adapting to the lower temperature, resulted in a shift in Q_p_ with time. The Q_p_ in the early exponential growth phase (days 3–6) of all 32 °C cultures was comparable or higher than the 37 °C cultures. Once the growth rate began to accelerate (days 6–9), the Q_p_ for the Ala cultures decreased to a value at or below the 37 °C Q_p_ whereas the Gly clones retained an elevated Q_p_ (Figure 4c). The maximum achievable antibody titers (<10% viable cells remaining in culture) obtained from the Ala clones were always lower when cultured at 32 °C than at 37 °C (Table 1). There was some clonal variation in the impact of the reduced temperature on the Ala clones, but this appeared to be linked with growth rate. The clone that exhibited the slowest growth at 37 °C (Ala-6, 3.3-fold decrease) was the most affected by the reduction in temperature whereas the fastest growing clone (Ala-174, 1.1-fold decrease) showed little change in expression. Both Gly clones showed a five-fold improvement in titer when cultured at the lower temperature. Therefore, the universal negative impact on Ala expression and positive impact on Gly expression indicates that the effect of reduced temperature on productivity was predominantly a function of the protein being expressed with minor effects from clonal variation.

### 2.3. Product Stability

**Figure 5.**
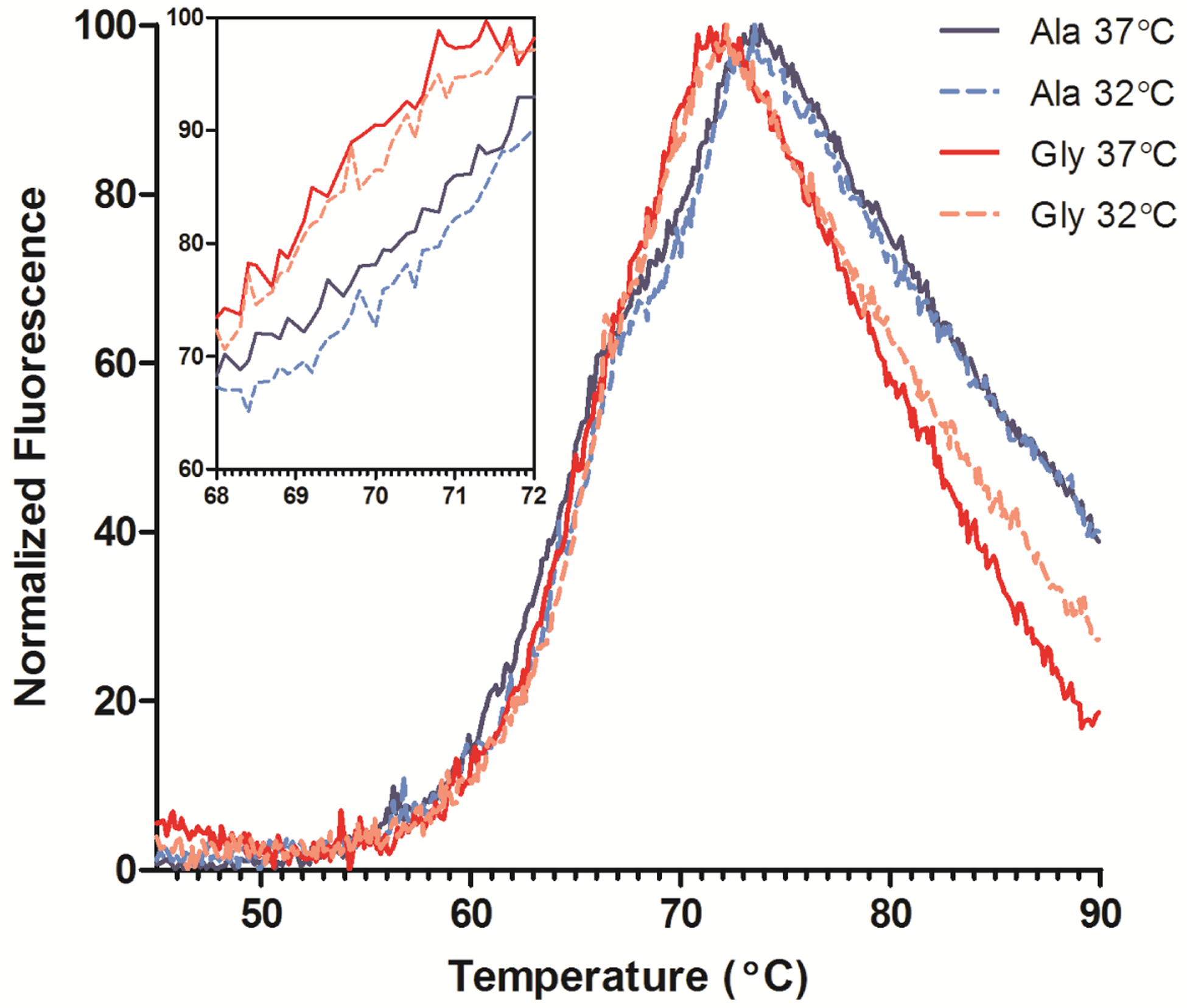
Differential scanning fluorimetry (DSF) profiles of purified Ala-138 and Gly-26 mAb cultured at 37 °C (solid lines) and 32 °C (dashed lines). The unfolding transitions for the C_H_2, C_H_3, and Fab domains are labeled. Each trace is the mean of four technical replicates. The inset graph shows detail over the Fab T_m_ range.

The stability of all samples was assessed using differential scanning fluorimetry (DSF). Instead of measuring the heat flux as in traditional calorimetric methods, DSF measures the fluorescence of an environmentally sensitive dye (SYPRO Orange) that binds to hydrophobic areas of protein [16]. As the protein unfolds, the increase in exposed hydrophobic residues coincides with an increase in dye binding, and therefore, fluorescence signal. The melting temperature (T_m_) is the temperature at which 50% of the protein is unfolded and can be estimated by finding the inflection point of each unfolding transition. For IgG_4_ antibodies, 65 °C is the expected melting temperature (T_m_) for a glycosylated Fc region and only one peak is observed due to cooperative unfolding of the C_H_2 and C_H_3 domains [17]. The humanized Fab domain can exhibit a wide range of T_m_’s typically between 60–80 °C [18]. The DSF traces indicated two transitions, one for the unfolding of the C_H_2 domain around 65 °C and the more intense Fab unfolding ~70 °C (Figure 5). The Ala and Gly variants exhibited similar C_H_2 melting profiles, but the Fab T_m_’s differed by ~1.5 °C. DSF was also performed on transiently-expressed His-tagged Fabs of the Ala and Gly variants to ensure the Fab T_m_ is independent of the Fc T_m_. The Gly Fab variant exhibited a T_m_ ~2 °C lower than the Ala Fab T_m_, which is consistent with the full-length results (data not shown). Therefore, the reduction in mAb stability could be attributed to the Ala to Gly mutation and not from any Fc domain influences. A comparison of antibody product obtained from cultures performed at 37 °C and 32 °C resulted in a slight increase in stability (~0.5 °C) of the antibodies from the low temperature cultures (Figure 5, inset); antibodies from both 32 °C cultures exhibited similar C_H_2 traces.

### 2.4. Circular Dichroism

**Figure 6.**
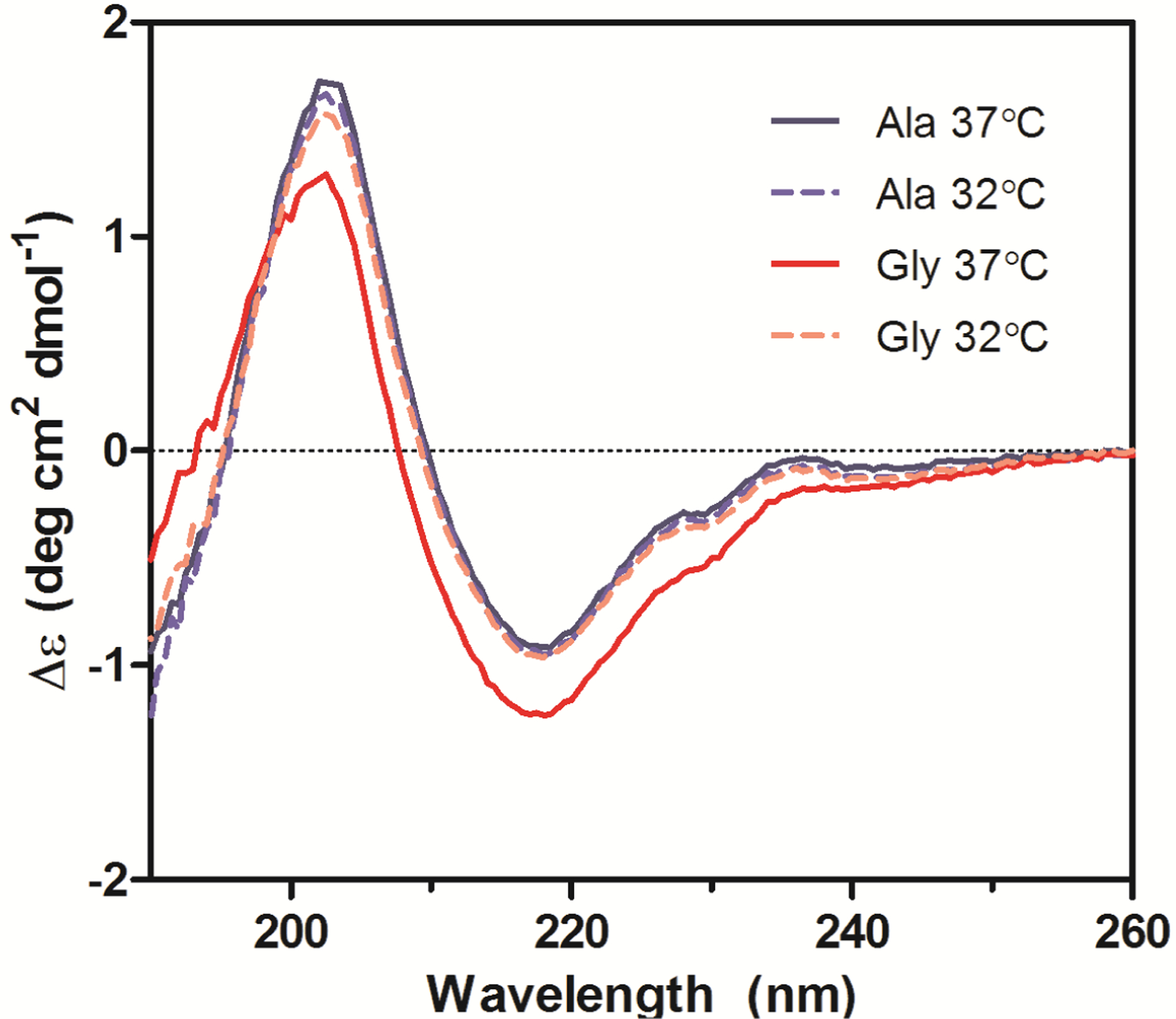
Far-UV CD spectra of purified Ala-138 and Gly-26 mAb cultured at 37 °C (solid lines) and 32 °C (dashed lines). Each trace is the average of three acquisitions and has had the phosphate buffer (20 mM, pH 8) signal subtracted. All measurements were performed at 10 °C.

**Figure 7.**
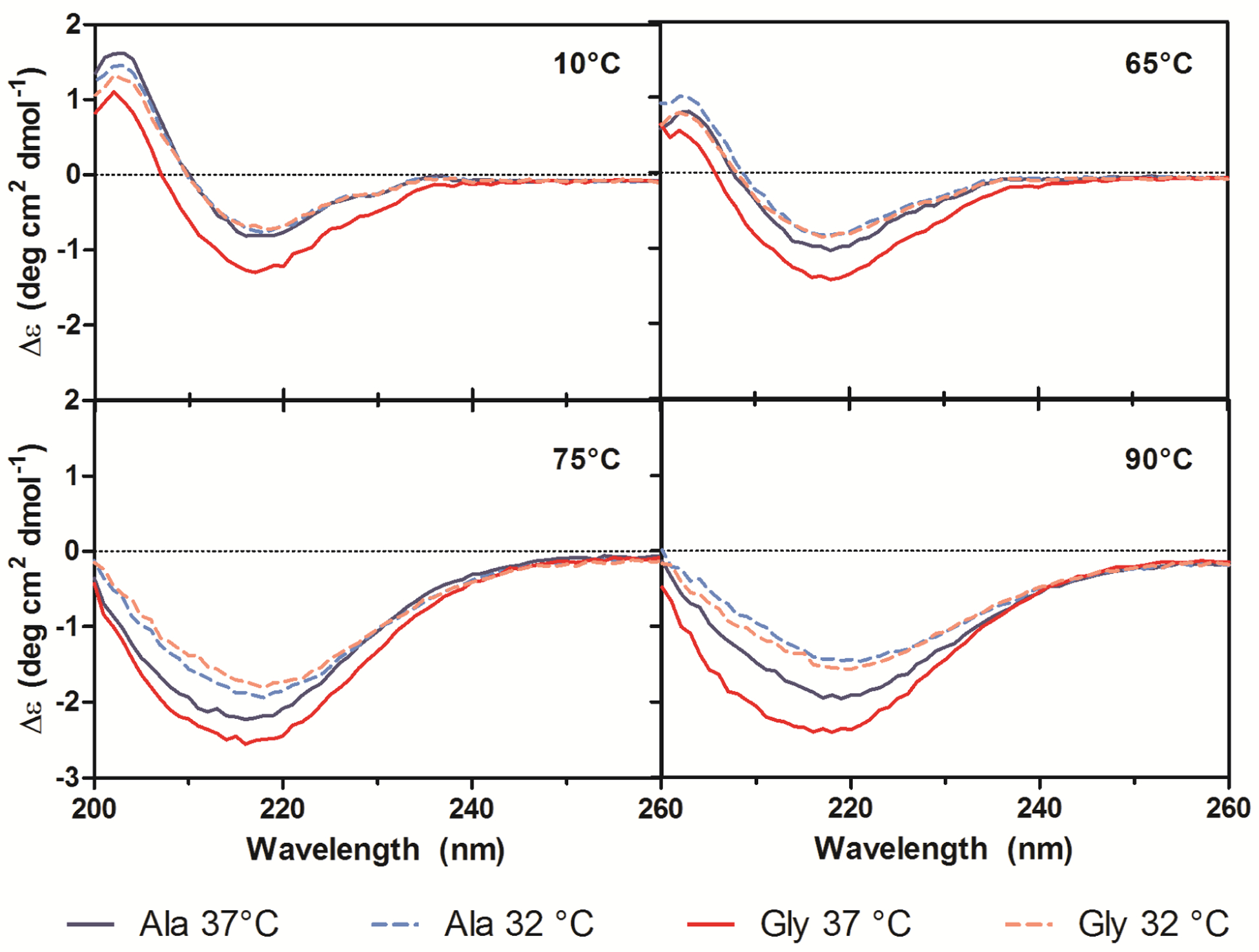
Far-UV CD traces at 10 °C, 65 °C, 75 °C, and 90 °C for purified Ala-138 (blue) and Gly-26 (red) mAb cultured at 37 °C (solid lines) and 32 °C (dashed lines). A heating rate of 1 °C/min was used.

Secondary structures of the purified Ala-138 and Gly-26 antibodies obtained from cultures at 37 °C and 32 °C were compared using far-UV CD. All spectra exhibited profiles characteristic of high β-sheet content proteins, which consist of a trough at ~217 nm and a peak at ~200 nm (Figure 6) [19–21]. A small shoulder at 230 nm was also present in all spectra and was attributed to contributions from aromatic side chain asymmetry. The CD traces for the Ala mAb cultured at both temperatures (37 °C and 32 °C) and the Gly mAb cultured at 32 °C were in good agreement with one another and exhibited similar behavior upon thermal denaturation. The spectrum of the Gly antibody purified from 37 °C culture deviated slightly from the other spectra. Monitoring the change in far-UV spectra as temperature was increased from 10 °C to 90 °C indicated little to no change in secondary structure until 65 °C (Figure 7). Between 65 °C and 75 °C, the trough at 217 nm became increasingly negative, widened, and exhibited a slight blueshift to 216 nm; the peak at 203 nm gradually disappeared. As the temperature was increased beyond 75 °C the trough amplitude decreased and the minima shifted to ~219 nm. Heating the 37 °C Gly sample resulted in similar behavior to all other samples up to 75 °C; no further changes in the far-UV spectra occurred above this temperature. Around 75 °C all spectra exhibited an increase in absorbance, coinciding with the formation of aggregates. The concomitant change in absorbance with temperature suggests that significant aggregation occurred in the 37 °C Gly antibody, but the differences in far-UV CD traces suggest that it may be a different type of aggregation than the other three samples. Further study is needed to characterize the aggregates formed upon thermal denaturation and determine if reduced culture temperature results in differential aggregation behavior.

The spectra obtained from the far-UV CD measurements were analyzed using the CDSSTR, CONTIN/LL, and SELCON3 programs contained in the CDPro analytical package. These algorithms attempt to fit the sample spectrum to an appropriate set of reference spectra compiled from proteins with well-characterized and diverse secondary structures [22]. All three algorithms gave similar fits of the spectra, which is required for a reliable analysis. The results of the analysis using the largest reference set available (56 proteins) indicated that all four samples contained ~45% β-sheet structures, 20% turns, 2.5% α-helical structures, and the remaining 32.5% did not fall under any of these classifications. The deviation of the 37 °C-cultured Gly sample is attributed to an increase in the amount of distorted α-helix compared to the other samples. The output from the CDPro analysis was compared to secondary structure assignment of the Ala variant Fab and an IgG_4_ Fc (Protein Data Bank entry: 1ADQ_A) crystal structure using the STRIDE algorithm (based on the DSSP algorithm) [23]. The CD analysis slightly underestimated the helical content, but was otherwise in agreement with the crystal structure data (Table 2).

To estimate the secondary structure content of the thermally denatured samples, the far-UV spectra acquired at 25 °C, 50 °C, 65 °C, 70 °C, 75 °C, 80 °C, and 90 °C were also analyzed by the CDPro package. The fits were only used to determine trends in the data because the temperature ramping was performed using a reduced acquisition range of 200–260 nm. Truncating the data below 200 nm decreases the reliability of the fit, especially for proteins with high β-sheet structure [19]. The signal from helical structures is much stronger than signal from β-sheets and therefore can result in difficulty estimating relative proportions of structure [21]. As temperature increased, the observed trends included a decrease in β-sheet and an increase in the fractions of α-helices, turns, and unordered protein. The analysis also suggested that the samples cultured at 32 °C retain more β-sheet structure and adopt less α-helix structure upon heating than their 37 °C counterparts. Again, the 37 °C-cultured Gly variant adopted a higher proportion of α-helix than all the other samples. The CONTIN/LL analysis was repeated on all samples at 10 °C and 75 °C to obtain more detail about the temperature-induced helix formation. A different set of reference proteins was used in the analysis which included the 3_10_-helix and the poly-L-proline II (PP2) helix motifs [24]. This analysis showed an increase in α-helix, 3_10_-helix, and turn content with a subsequent decrease in fractions of β-sheet, PP2, and unordered protein (Table 3). The same trends were observed between samples, with the 37 °C cultures adopting more helical structure and the 32 °C cultures retaining more β-sheet upon thermal denaturation.

The increase in α-helix content of IgGs upon thermal denaturation is supported by previous experimental work. One study attributed the increase in α-helix content to hydrogen bonding between aggregate interfaces [25]. Other proteins with high β-sheet content, such as TNF-α, have shown an increased propensity for α-helix formation upon heating [26]. It was thought that the loss of tertiary structure removes critical long-range interactions, and new short-range interactions result in adoption of intermediate structures containing more α-helix structure. Here, both protein sequence and culture incubation temperature appear to affect the adopted secondary structure upon thermal denaturation. Using a reduced culture temperature of 32 °C appeared to allow the antibodies to adopt a β-sheet structure that was more resistant to heat-induced α-helix formation. The same resistance of the 32 °C cultures to α-helix formation was also observed in isothermal denaturation at 65 °C (data not shown). In the case of the Gly variant, reduced culture temperature had a more dramatic effect and seemed to prevent the mutation-induced formation of α-helix. The reduced culture temperature only had a modest effect on thermal stability of the molecules, but may have more important consequences for aggregation propensity. Further work is needed to elucidate the exact changes to secondary structure imposed by reduced temperature culture.

**Figure 8.**
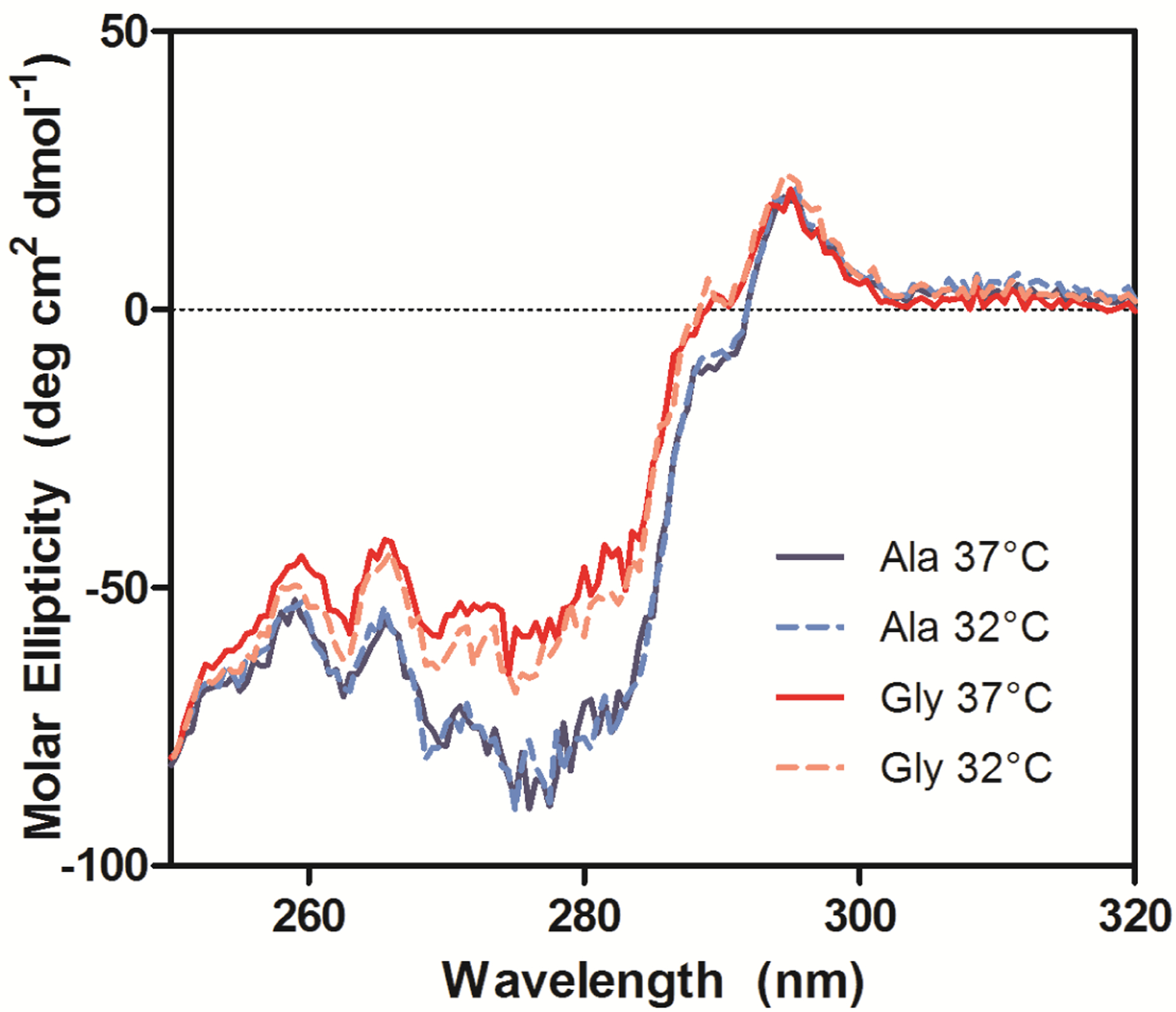
Near-UV CD spectra of purified Ala-138 and Gly-26 mAb cultured at 37 °C (solid lines) and 32 °C (dashed lines). Each trace is the average of three acquisitions and has had the phosphate buffer (20 mM, pH 8) signal subtracted.

The near-UV CD spectra, which were used to compare the tertiary conformation of the samples, indicated that the Ala mAbs were in the same conformation, but the Gly mAbs not only differed from the Ala samples, but also from each other (Figure 8). The most significant deviations in the spectra occurred between 270–295 nm, which is mainly comprised of Tyr (270–290 nm) and Trp (280–300 nm) signals [20]. Analysis of the antibody crystal structure indicated the majority of these residues are either buried in the hydrophobic core of domains or present at the HC/LC interface. The amino acid occupying Kabat position 49 potentially interacts with or impacts the conformation of two Trp and two Tyr residues. The side chain of Ala interacts with the buried Trp-36 and Tyr-60 residues whereas the Trp-47 and Tyr-59 reside on the surface (Figure 1). Trp-47 is a highly conserved residue that directly interacts with the V_L_ interface [27]. Replacing Ala with Gly may eliminate several interactions within the hydrophobic core of the V_H_ domain that could result in the observed alternate conformation.

The combined biophysical data indicates that the Ala to Gly mutation at Kabat position 49 resulted in a change in the V_H_ conformation, which coincided with decreased expression in CHO cells. Introducing a Gly residue to a β sheet is known to be intrinsically destabilizing [28]; stability can be reinstated by cross-strand pairing with an aromatic residue in a process known as aromatic rescue [29]. The aromatic partner can adopt a rare conformation to “shield” the Gly, which is impossible with any other residues due to the steric restrictions imposed by all other amino acid side chains [30,31]. If aromatic shielding occurs in the Gly antibody, then the side chain of its aromatic neighbors could lose their native interactions in the V_H_ hydrophobic core or the HC/LC interface. The increased proportion of α-helix observed in the 37 °C-cultured Gly construct could be the result of an intermediate conformation that remains upon assembly with the LC. If the HC/LC variable region interface is disturbed, the interface may be more solvent-accessible, causing local burying of hydrophobic residues. This may account for the differences in near UV CD spectra. Decreasing the culture temperature results in the Gly variant adopting the same secondary structure content as the Ala variant, potentially through mechanisms such as aromatic shielding or increased contact time between the HC and LC, but does not fully rescue the tertiary conformation. This result is expected, as the changes required to stabilize the secondary structure would compromise interactions that dictate the tertiary conformation.

## 3. Experimental Section

### 3.1 Cell Culture

The generation of recombinant IgG-producing stable cell lines used in this study has been described previously [12]. CHO-S cells (Invitrogen, Paisley, UK) were cultured in CDCHO medium supplemented with 4 mM GlutaMax; all stable clones were cultured in CDCHO medium containing 50 μM methionine sulfoximine (MSX). All cells were subcultured every 3–4 days in Erlenmeyer shake flasks with an inoculum viable cell density (VCD) of 3 × 10^5^ cells/mL. Flasks were incubated at 37 °C in 8% CO_2_ with 140 rpm shaking. All cell counts were performed using a Cedex A520 automated cell counter (Innovatis, Bielefeld, Germany). Antibody concentrations were measured using an Octet QK (ForteBio, Menlo Park, CA, USA) with Protein A biosensors as described below. Specific productivity, *Q*_*p*_ (pg/cell/day), was calculated during exponential growth phase using the following equation:

(1) $$Q_{p}=\frac{10\cdot \text{ln}\left(\frac{n_{t}}{n_{0}}\right)\Delta P}{\left(n_{t}-n_{0}\right)t}$$

where *t* is the elapsed culture time in days, *n*_*0*_ and *n*_*t*_ are the viable cell densities (10^6^ cells/mL) at the start of the culture and time *t* respectively, and *ΔP* is the change in antibody titer (μg/mL) between the start and end of culture.

### 3.2. Plasmids

Double gene vectors (DGVs) containing the heavy chain (HC) and light chain (LC) genes under individual control of the human CMV promoter were used for transient experiments. All plasmids were prepared using MiniPrep or MaxiPrep kits (Qiagen, West Sussex, UK). DNA concentration was measured using a Nanodrop 1000 spectrophotometer (NanoDrop Products, Wilmington, DE, USA); DNA was considered pure if the A260/A280 ratio ≥ 1.8.

### 3.3. Transient Transfection

All transient transfections were performed with CHO-S cells using an Amaxa Nucleofector II (Nucleofector Kit V) (Lonza Biologics, Slough, UK). The kit was used according to the manufacturers’ instructions. For each sample, 1 × 10^7^ cells were transfected with 5 μg DNA using Amaxa program U-024. The transfected samples were diluted in 20 mL of CDCHO medium supplemented with 4 mM GlutaMax. Flasks were incubated at 37 °C or 32 °C in 8% CO_2_ and shaken at 140 rpm.

### 3.4. Quantitative Real-Time PCR

Total RNA was isolated from 1 × 10^6^ cells in mid-exponential phase using a RNeasy kit (Qiagen, West Sussex, UK). The concentration and purity of the extracted RNA was determined using a Nanodrop 1000 spectrophotometer (NanoDrop Products, Wilmington, DE, USA) and was considered pure if the A_260_/A_280_ ratio was > 2.0. Up to 2 μg of total RNA was used to generate cDNA using a High Capacity cDNA Reverse Transcription Kit (Applied Biosystems, Foster City, CA, USA). The cDNA was diluted 1:100 with ultrapure DNase, RNase-free water. TaqMan master mix solutions were prepared by mixing 5 μL of TaqMan Gene Expression Master Mix (Applied Biosystems, Foster City, CA, USA) with 0.5 μL of 20X TaqMan custom gene expression assay per sample. The TaqMan primer and probe sequences are listed in Table 4. Triplicate qPCR reactions were loaded onto a 384-well plate containing 4.5 μL of sample and 5.5 μL of TaqMan master mix. qPCR reactions were performed on an ABI 7900HT Fast Real-Time PCR system using an initial 10 min activation step at 95 °C followed by 40 cycles of 15 s denaturation at 95 °C and 1 min anneal/extend at 60 °C. Relative quantitation data analysis was performed using the comparative quantification method, ΔΔC_t_, with GAPDH as the endogenous reference. The amplification efficiencies of the genes of interest were comparable over the input cDNA concentrations used in this study. The calibrator used is specified in the appropriate results section.

### 3.5. Antibody Assays

Antibody titers were measured using an Octet QK (ForteBio, Menlo Park, CA, USA) with Protein A Biosensors according to the manufacturer’s instructions. The appropriate number of biosensors were prewet in 200 μL media or lysis buffer for a minimum of 5 min prior to use in a 96-well plate. The samples for analysis were prepared by adding 200 μL to a black, flat-bottomed 96-well plate. The analysis was performed at 30 °C with a 2-min run time and 200 rpm plate agitation. Final antibody titers were compared to a standard IgG_4_ curve over a working range of 1 to 700 μg/mL.

### 3.6. Protein Purification

Supernatants containing the antibody product from stable cell lines were purified on a Mab Select SuRe Protein A column using an AKTA Prime system (GE Healthcare, Little Chalfont, UK). The column was equilibrated with 5 column volumes (CVs) of PBS, followed by loading of the supernatant. Column loading was monitored by measuring the absorbance at 280 nm. The loaded column was rinsed with 5 CVs of PBS or until the A_280_ baseline returned to zero. The bound antibody was then eluted with 0.1 M sodium citrate buffer, pH 3. Eluted fractions were immediately neutralized with 1/4 of the fraction volume of 2 M Tris·HCl, pH 8. The purified mAbs were buffer exchanged into phosphate buffered saline (PBS), pH 7.4 or 20 mM sodium phosphate, pH 8 using PD-10 desalting columns (GE Healthcare, Little Chalfont, UK). The mAb concentration was measured by OD_280_ using a calculated extinction coefficient [32,33].

### 3.7. Differential Scanning Fluorimetry

Purified mAb was diluted to 0.1 mg/mL with PBS. A 30X fluorescent dye solution was made by mixing 6 μL 5000X Sypro Orange dye (Invitrogen) with 994 μL PBS. Samples were prepared by mixing 45 μL diluted mAb with 5 μL of 30X dye and adding 10 μL of the mixture to a 384-well plate in quadruplicate. The plate was sealed with optical film and analyzed on the ABI 7900HT Fast system using an initial 5 min hold at 20 °C, ramp to 99 °C at 1 °C/min, and a final hold for 20 min at 99 °C. Data was analyzed using in-house Excel macros.

### 3.8. Circular Dichroism Spectroscopy

Circular dichroism (CD) spectroscopy was performed using a Chirascan Spectrometer (Applied Photophysics, Leatherhead, UK). For measurements in the far-UV region (190–260 nm) samples were prepared at 0.5 mg/mL in 20 mM sodium phosphate buffer, pH 8, and spectra obtained using a 0.5-mm-pathlength rectangular quartz cuvette. For measurements in the near-UV region (250–320 nm), samples were prepared at 1 mg/mL in phosphate buffer and added to a 1-cm-pathlength cuvette. Scans were performed in triplicate at 10 °C using a 1-s acquisition time and 0.5-nm step size. Any background signal was removed by subtracting the phosphate-buffer spectrum. The far-UV data was converted from the machine units of ellipticity (*θ*, mdeg) to per residue delta epsilon (Δ*ε*, deg cm^2^ dmol^−1^) using:

(2) $$\Delta \varepsilon =\frac{\theta \cdot MRW}{10\cdot C\cdot l\cdot 3298}=\frac{\left[\theta \right]}{3298}$$

where *C* is the protein concentration in mg/mL, *MRW* is the mean residue molecular weight (protein molecular weight/number amino acids (*N*) in the sequence), and *l* is the cuvette pathlength in cm. The near-UV data was converted from ellipticity to molar ellipticity ([*θ*], deg cm^2^ dmol^−1^). Temperature-ramped far-UV CD measurements were acquired between 200–260 nm using a 1 °C/min ramp rate. To keep spectrum acquisition less than 1 min, the time-per-point was reduced to 0.4 s and a 1 nm step size was used. Secondary structure analysis was performed using the CDPro software package [34].

## 4. Conclusions

The use of sub-physiological temperatures to alter recombinant gene expression is well documented in literature and has produced varied results [4]. However, closer analysis of reported data shows that mild hypothermia (31–33 °C) is successful at increasing specific and/or volumetric productivity in CHO cells, but the degree of enhancement varies with the protein being expressed. This enhancement appears to falter when temperatures at or below 30 °C are applied or when hybridoma cell lines are used for expression.

Here we have investigated the effect of culture temperature on expression of antibody variants that exhibit intrinsic differential expression in CHO cells. Decreasing the temperature from 37 °C to 32 °C increased the specific productivity (particularly for the Gly variant) and modestly increased the volumetric productivity of cells transiently expressing both antibody variants due to the growth arrest. Interestingly, increases in mRNA levels were only seen in the Gly variant. When this technique was applied to stable cell lines that have successfully integrated transgene DNA, there was no enhancement for clones producing the Ala variant, but improvement in expression of the Gly variant occurred. Analysis of the biophysical properties of the antibodies purified from 37 °C cultures indicated that the Fab fragment of the Ala variant is intrinsically more stable than the Gly variant and that they exhibited different tertiary configurations. Decreasing the culture temperature to 32 °C appeared to improve protein folding fidelity. This resulted in a slight increase in stability for both constructs, and permitted the Gly variant to adopt a similar configuration to that of the Ala variant. The change in the tertiary configuration of the Gly mAb at lower temperatures coincided with an increase in expression. The Ala mAb did not show any change in secondary or tertiary structure at lower temperatures, and consequently, no improvement in stable expression was observed. These results suggest that reduced incubation temperatures caused protein-level changes, in addition to the observed message-level changes, which resulted in enhanced recombinant antibody expression of the Gly variant.

To our knowledge, this is the first study to show a differential effect of reduced temperature on expression of closely related antibody variants and to correlate temperature-induced change in expression with the biophysical properties of the purified antibody. This study supports sequence design as the critical step in maximizing recombinant antibody expression in CHO cells, but suggests that decreased temperature may be used as a potential tool to rescue expression in proteins where sequence engineering strategies are not possible. The observed differences in protein structure found in this study suggest reduced culture temperature may have a greater influence on product quality than previously thought and therefore warrants further investigation.

## Figures and Tables

**Table 1.** Effect of culture temperature on antibody expression.

Construct	Max. Titer at 37 °C (μg/mL)	Max. Titer at 32 °C (μg/mL)	Titer 32 °C/37 °C
**Ala-174**	7.42	6.72	0.91
**Ala-6**	814	247	0.30
**Ala-138**	1124	547	0.49
**Ala-187**	848	389	0.46
**Gly-75**	3.31	14.9	4.50
**Gly-26**	12.2	63.3	5.19

**Table 2.** Secondary structure estimation from CD data using the CDPro software package.

Algorithm	Sample	α_R_	α_D_	α_T_	β_R_	β_D_	β_T_	T	U	RMSD	NRMSD
	Ala Fab X-Ray	-	-	0.060	-	-	0.485	0.239	0.216	-	-
	Ala Fab + Fc X-Ray	-	-	0.071	-	-	0.490	0.232	0.207	-	-
**CONTIN/LL**	Ala-138 37 °C	0.000	0.024	0.024	0.298	0.149	0.447	0.198	0.331	0.066	0.083
	Ala-138 32 °C	0.000	0.027	0.027	0.290	0.148	0.438	0.200	0.335	0.055	0.070
	Gly-26 37 °C	0.001	0.031	0.032	0.292	0.142	0.434	0.207	0.327	0.058	0.074
	Gly-26 32 °C	0.000	0.025	0.025	0.298	0.148	0.446	0.201	0.328	0.058	0.076
**CDSSTR**	Ala-138 37 °C	0.000	0.001	0.001	0.300	0.150	0.450	0.231	0.308	0.099	0.126
	Ala-138 32 °C	0.000	0.006	0.006	0.306	0.143	0.449	0.238	0.300	0.129	0.165
	Gly-26 37 °C	0.000	0.016	0.016	0.297	0.140	0.437	0.236	0.308	0.085	0.108
	Gly-26 32 °C	0.000	0.005	0.005	0.303	0.149	0.452	0.234	0.299	0.092	0.121
**SELCON3**	Ala-138 37°C	0.000	0.000	0.000	0.246	0.154	0.400	0.237	0.354	0.224	0.284
	Ala-138 32 °C	0.000	0.000	0.000	0.240	0.152	0.392	0.244	0.365	0.215	0.274
	Gly-26 37 °C	0.000	0.019	0.019	0.253	0.146	0.399	0.232	0.353	0.172	0.220
	Gly-26 32 °C	0.000	0.004	0.004	0.243	0.151	0.394	0.235	0.343	0.237	0.314

Abbreviations: α_R_, regular α-helix; α_D,_ distorted α-helix; α_T,_ total α-helix; β_R,_ regular β-sheet; β_D,_ distorted β-sheet; β_T,_ total β-sheet; T, turns; U, unordered protein; RMSD, root mean square deviation; NRMSD, normalized root mean square deviation.

**Table 3.** Secondary structure estimation from CD data of native and denatured antibodies.

Temperature	Sample	α-helix	3_10_-helix	B-sheet	Turn	PP2	Unordered	RMSD	NRMSD
**10 °C**	Ala-138 37 °C	0.001	0.013	0.351	0.106	0.105	0.425	0.078	0.129
	Ala-138 32 °C	0.002	0.013	0.344	0.108	0.107	0.426	0.057	0.105
	Gly-26 37 °C	0.018	0.023	0.336	0.114	0.095	0.414	0.081	0.125
	Gly-26 32 °C	0.002	0.013	0.338	0.110	0.109	0.428	0.061	0.123
**75 °C**	Ala-138 37 °C	0.114	0.045	0.243	0.132	0.079	0.386	0.025	0.020
	Ala-138 32 °C	0.089	0.038	0.260	0.138	0.080	0.395	0.029	0.027
	Gly-26 37 °C	0.141	0.049	0.226	0.135	0.073	0.374	0.031	0.021
	Gly-26 32 °C	0.080	0.038	0.267	0.139	0.081	0.396	0.036	0.036

**Table 4.** TaqMan primer/probe sequences used in qPCR reactions.

Target	Primer	Sequence (5’→3’)
**Human constant κ (Light Chain)**	Forward	AAAGTACAGTGGAAGGTGGATAACG
	Reverse	CTTGCTGTCCTGCTCTGTGA
	Probe	CCAATCGGGTAACTCC
**Human γ4 (Heavy Chain)**	Forward	CCCAAGGACACTCTCATGATCTC
	Reverse	CCATCCACGTACCAGTTGAACT
	Probe	ACGCACGTGACCTCAG
**Glutamine Synthetase**	Forward	TGCCCAGTGGGAATTCCAAATAG
	Reverse	GGGCCACCCAGAGATGATC
	Probe	CCATGCGGATTCCTT
**Hamster BiP**	Forward	GGTGGGTCTACTCGGATTCC
	Reverse	CTCCTTGCCATTGAAGAACTCTTTC
	Probe	ACCAGCTGCTGAATCT
**Hamster GAPDH**	Forward	CTGCCACCCAGAAGACTGT
	Reverse	GTGGATGCAGGGATGATGTTCT
	Probe	ATCACGCCACAGCTTT
